# Knowledge map and emerging trends of oxidative stress in wound healing: A bibliometric analysis from 2000 to 2023

**DOI:** 10.1097/MD.0000000000039970

**Published:** 2025-03-07

**Authors:** Fan Bu, Kai Yu, Jinnan Wang, Li Rong, Qiaoyu Li

**Affiliations:** a Department of Otolaryngology, Head, Neck and Plastic Surgery, Honghui Hospital, Xi’an Jiaotong University, Xian, China; b Department of Plastic and Aesthetic Surgery, Honghui Hospital, Xi’an Jiaotong University, Xian, China; c Department of Plastic and Aesthetic Surgery, The First Hospital of Jilin University, Changchun, China; d Department of Urology, The First Hospital of Jilin University Changchun, Changchun, China; e China Department of Urology, The First Hospital of Jilin University, Changchun, China; f Department of Thyroid Surgery, The First Hospital of Jilin University, Changchun, China.

**Keywords:** Bibliometric analysis, CiteSpace, oxidative stress, repair, VOSviewer, Wound

## Abstract

The skin’s integrity is vulnerable to external elements that can induce injuries, leading to wound formation. It’s crucial to comprehend wound healing processes to protect the body when this protective barrier is compromised. Over the last 2 decades, there has been considerable progress in understanding delayed wound healing, with a focus on the mechanisms and microenvironmental factors involved. The connection between oxidative stress and wound healing has recently gained attention, emphasizing the need for in-depth analysis to propel further advancements and interventions in this area. Despite these advancements, there remains a noticeable void in the literature concerning the application of scientometric methods to systematically examine the progression of wound healing research. Additionally, a comprehensive assessment of the research output and effectiveness of various researchers and institutions in this field is lacking. To address these gaps, we analyzed data from the Web of Science Core Collection from January 1, 2000, to December 31, 2023, utilizing relevant keywords. Using CiteSpace, we created visual maps that depict the evolution and structure of keyword clusters, and both CiteSpace and VOSviewer were used to evaluate the performance of research networks across different countries, institutions, and authors. This data was methodically analyzed. The leading institution in this field is the Chinese Academy of Medical Sciences. The key researchers are Bekeschus, Sander; Li, Yang; Bi, Yang; Fan, Daidi; and Zhang, Yu. Our software analysis reviewed 3025 studies, revealing 19 co-citation clusters that highlight current trends in research on oxidative stress and wound healing. Prominent journals, leading institutions, and key researchers were identified. Key emerging research directions include studying the mechanisms linking oxidative stress to wound healing, exploring the use of antioxidant substances in wound dressings, and investigating how nanomaterials in dressings can influence oxidative stress. These focal points emphasize the significance of understanding oxidative stress’s impact on wound healing and investigating new methods to enhance therapeutic efficacy. This comprehensive approach not only fills a gap in the current literature but also sets the stage for future research endeavors in this crucial area of health science.

## 1. Introduction

The skin, recognized as the largest organ of the human body, is situated on its outermost layer. It serves several critical functions, including sensing the external environment and regulating body temperature. Crucially, the skin’s barrier function, which is essential for protecting the body, depends on maintaining its integrity and continuity. This barrier not only shields the internal organs from environmental threats but also helps to prevent water loss and ward off pathogens.^[1,2]^ Trauma compromises the integrity of the skin, leading to the formation of a wound.^[3]^ The human body has innate capabilities for wound repair, and the processes involved in wound healing are intricate.^[4–7]^ Several factors may impede this healing process, including oxidative stress, the presence of foreign bodies, reduced blood flow (ischemia), infections, aging, and poor nutrition.^[8]^ These adverse influences can lead to complications such as infections, pain, and scarring during the healing process.^[9]^ Given the intimate relationship between oxidative stress and various harmful factors, oxidative stress plays a pivotal role in the process of wound healing. While moderate levels of oxidative stress are thought to enhance immune responses^[10]^ and facilitate wound healing, an excessive presence of oxidative stress can impede the healing process and disturb the internal environment’s homeostasis.^[10]^ Hence, regulating an optimal level of oxidative stress can accelerate wound healing to a certain extent.^[11]^

Our main objective is to provide a detailed description of how research on oxidative stress reactions in skin wound healing has evolved over time. We aim to identify the evolution of key research topics by using co-citation networks and emerging keywords, thereby providing new research directions for future studies in this field. Additionally, our secondary objective is to offer metrics for the research network – encompassing countries, institutions, authors, and journals – to relevant researchers. This analysis will help detect research abundance, gaps, emerging trends, biases, and limitations.

Over the recent years, there has been a notable surge in research focusing on the impact of oxidative stress on wound healing. The exponential growth in the number of published articles on this topic can be attributed to various factors:

### 1.1. Oxidative stress alters the microenvironment of wound healing, thereby affecting the process of wound healing

The fundamental process of wound healing relies on inflammation and tissue regeneration, with debridement and repair serving as essential stages. Despite the evolution of tissue regenerative capabilities, systemic and local factors still significantly influence the process. Both systemic and local factors play a role in influencing regenerative repair. Systemic factors, such as patient age and nutritional status, alongside local factors, including the wound’s surrounding environment encompassing reactive oxygen species, pH, molecules, and enzymes, impact tissue regeneration. Disrupted oxidative stress reactions can result in pH alterations, changes in enzyme activity,^[12]^ and the generation of inflammatory substances,^[13]^ thereby underscoring the critical role of oxidative stress in wound healing. As adjusting systemic factors can be challenging, regulating local oxidative stress to enhance the local wound environment has emerged as a strategy to facilitate faster and improved wound healing.^[14]^

### 1.2. Oxidative stress exerts a notable influence on the fundamental structure of living organisms and their repair mechanisms

Oxidative stress delineates the excessive generation of reactive oxygen species (ROS) or metabolic disturbances surpassing the inherent antioxidant defense system’s capability to eliminate them. The surplus ROS mediate the oxidation of biomolecules, culminating in cell lipid peroxidation and harm to lysosomes and mitochondria. While a modest quantity of reactive oxygen species is essential for thwarting external harm, heightened oxidative stress and feeble antioxidant capacity are pivotal factors contributing to delayed wound healing.^[15,16]^ ROS overexpression can instigate oxidative stress, inflicting harm on cell lipids, proteins, and DNA, thereby prompting cell demise and triggering detrimental processes like necrosis, inflammation, and fibrotic scarring.^[17]^ Hydrogen peroxide and superoxide can reflect the extent of oxidative stress, with elevated levels indicating a robust oxidative stress reaction.^[18]^ Intermediate byproducts engendered by oxidative stress, such as hydrogen peroxide and peroxynitrite, can regulate cell signal transduction and evoke cytotoxic effects, assaulting and impairing biomolecules in the body, such as DNA and proteins,^[19]^ consequently impacting cell longevity. Research has associated these byproducts with delayed wound healing. Thus, in the investigation of delayed wound healing, diminishing oxidative stress at the wound site using various methodologies has emerged as a paramount focus for averting delayed wound healing.

### 1.3. The contemporary research focus on investigating the influence of oxidative stress on wound healing has been consistently progressing

One commonly utilized approach to counteracting oxidative stress is through the application of antioxidants. Given that wound dressings are in direct contact with the wound, incorporating antioxidants directly into them is a practical strategy to combat oxidative stress. The primary functions of wound dressings entail upholding cleanliness at the wound site, curbing bacterial proliferation,^[20]^ retaining moisture to impede excessive water loss,^[21]^ lowering oxidative stress levels at the wound site, and expediting wound recovery. These attributes collectively foster an ideal microenvironment for effective wound healing. It is a challenge to find a single wound dressing that encompasses all desired attributes, such as high biocompatibility, biodegradability, and mechanical robustness.^[22]^ Nevertheless, integrating nanomaterials with wound dressings due to their versatile properties can help achieve these sought-after characteristics. By infusing chemical components like antioxidants into wound dressings and incorporating nanotechnology, researchers can augment oxidative stress reduction and facilitate quicker, enhanced wound healing.^[23]^ This progressive research direction aligns with the advancement of wound dressings within the realm of biomedical engineering.

Over the years, numerous scholars have made significant progress in investigating oxidative stress and its implications on wound healing. The exploration of wound healing enhancement through such dressing methods has been extensively explored. However, these investigations lack systematic evaluation, with only a sparse number of literature-based examinations delving into the mechanisms of wound recovery. Leveraging bibliometric analysis tools, we conducted a comprehensive review of these studies, systematically tracing the research trajectory over time. This visual representation and analysis not only offer researchers and clinicians a clearer understanding of the topic’s research landscape but also aid in pinpointing research abundance, gaps, trends, and potential influential factors (such as publication year and methodological variances). It also sheds light on potential biases or limitations. The iterative process of organizing this dataset enhances comprehension of the subject, facilitating subsequent reading and analysis, especially in identifying research foundations and trending areas. Furthermore, it allows for examination of research institutions, scholars, key journals, and preference trends, thereby contributing positively to the advancement of research on oxidative stress at wound sites. It additionally offers insights and inspiration for individuals engaged in wound healing research and treatment, fostering international collaboration and technological progress in the field.

## 2. Methods

### 2.1. Scientometric analysis

Bibliometric studies aim to describe and visualize the complex relationships within a knowledge cluster and explore the structure and evolution of scientific knowledge.^[24]^ In our study, we used the Web of Science Core Collection (WoSCC) to retrieve articles on the relationship between wound healing and oxidative stress from 2000 to the end of 2023, and we extracted data from PubMed as well. Search terms were refined to optimize search logic, using TS = (wound repair) and TS = (oxidative stress). The primary terms included “skin repair,” “wound healing,” and “oxidative stress.” The search was limited to papers and reviews published in English.

### 2.2. Search strategy and data collection

Our data collection was primarily conducted through the WoSCC, chosen for its comprehensive coverage in scientific bibliometric analysis. Search terms combined medical subject headings and free terms, with a focus on “Injuries” or “Wound,” restricted to “Science Citation Index Expanded,” and publication types such as “Original Research” or “Review.” The complete records of cited references published before December 31, 2023, were extracted into a tagged delimited plain-text file. We used CiteSpace to remove duplicates, resulting in 3025 relevant articles (Supplementary Figure S1, http://links.lww.com/MD/O154).

The WoSCC data served as the foundation for further analysis using CiteSpace and VOSviewer.

### 2.3. Objectives

Our primary goal is to describe the evolution of research on oxidative stress in skin wound healing, identifying key topics and suggesting future research directions. Additionally, we aim to provide metrics for the research network, helping identify trends, gaps, and biases.

### 2.4. Data analysis

We utilized VOSviewer (version 1.6.18) and CiteSpace (version 6.2.2) for our analysis. The measurement units used include authors, journals, references, countries, institutions, and keywords. Network visualizations used in scientometric mapping include directed and undirected graphs. The direct citation network is a directed graph, while co-citation and co-occurrence networks are undirected graphs. Bibliometric results include citation counts, co-citation, and cooccurrence.

The count refers to the number of times a publication is cited by other papers, while co-citation is defined as the number of times two articles are simultaneously cited by the paper. Co-citation networks are particularly suitable for systematic reviews, as co-citation links can reveal how clusters develop independently of the original publications. Co-occurrence networks represent the graphical representation of the frequency with which variables appear together. The mapping results of the system are network and co-citation (or co-occurrence) clusters. CiteSpace’s automatic cluster labeling and summarization enhance the interpretation of these clusters.

CiteSpace provides various significance indicators, including temporal indicators (such as citation bursts), structural indicators (such as betweenness centrality, clustering score, and modularity score), and their combination (i.e., sigma). Betweenness centrality, measured according to Freeman’s metric, quantifies the number of times a node lies on the shortest path between other nodes. Nodes with high betweenness often connect different clusters and are considered key hubs. Burstiness measures the rate of change. The burstiness of an entity’s frequency over time indicates specific durations when the frequency undergoes rapid changes, thus identifying emergent items. CiteSpace combines betweenness centrality as a structural attribute with citation bursts as a temporal attribute using a measure called sigma. Sigma is calculated as (centrality + 1) × burstiness, and a higher value indicates greater potential impact of the work. The modularity (Q score) of a network measures the extent to which the network can be divided into modules or clusters, while the silhouette (S score) is a method to assess the consistency of interpretation and validation within the data clusters. The Q score ranges from 0 to +1, while the S score ranges from −1 to +1. For both indicators, scores close to +1 indicate the best clustering models. When the Q value exceeds 0.3, the clustering structure is considered significant, with higher values indicating a better network structure. The silhouette coefficient exceeding 0.3, 0.5, or 0.7 indicates a network that is considered to be uniform, reasonable, or highly reliable, respectively. However, a silhouette value of 1 may indicate relative isolation of the corresponding cluster.

When examining the burstiness of published articles, it indicates a specific article is associated with a sudden increase in citations. We excluded references involving descriptive and categorization of mental disorders (e.g., DSM, ICD-10) from the model. Where appropriate, we merged redundant nodes. The impact factor of the journals included was retrieved from the 2023 Journal Citation Reports (data extracted from the plain-text file from WoSCC).

VOSviewer (version 1.6.18) was used to obtain the most cited journal network and cooccurring author keyword network visualizations. CiteSpace (version 6.2.2) was used for extracting collaboration networks (across countries and institutions), co-citation analysis (cocited author and co-cited reference clusters), and co-occurrence analysis (co-occurring author keyword network). Burst analysis was performed using CiteSpace for all measurement units. The g-index is an author-level indicator based on the distribution of received citations, mitigating the bias towards highly cited papers, just like the h-index. The g-index is used for all calculations. Importantly, the inflation value of the g-index helps give credit to lowly cited or uncited papers while penalizing highly cited ones, making it more relevant to co-citation analysis. CiteSpace also optimized time slicing by removing empty time intervals. CiteSpace parameters are provided in Supplementary Figure S1, http://links.lww.com/MD/O154. The scaling factor k for all analyses was set to 25.

## 3. Results

**Figure 1.**
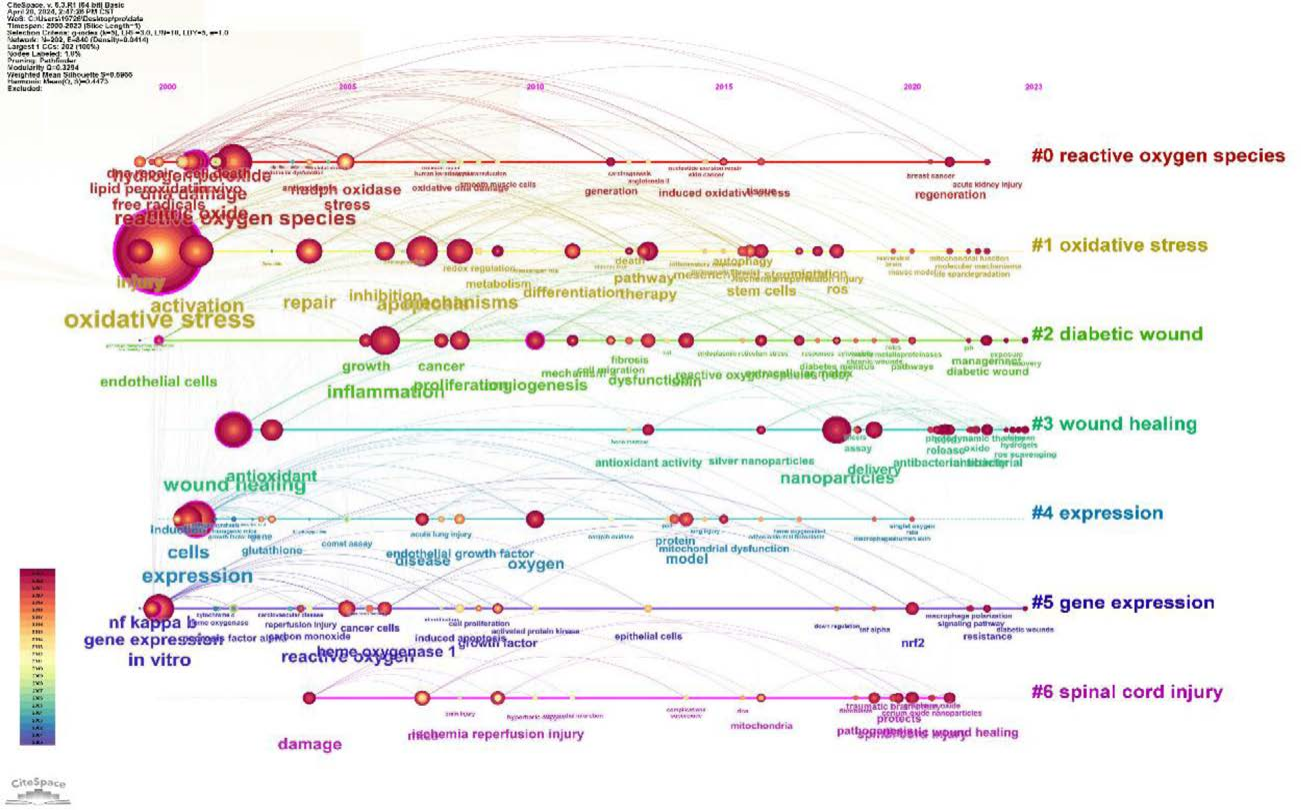
The change in the trend of co-occurrence with citations over time.

**Figure 2.**
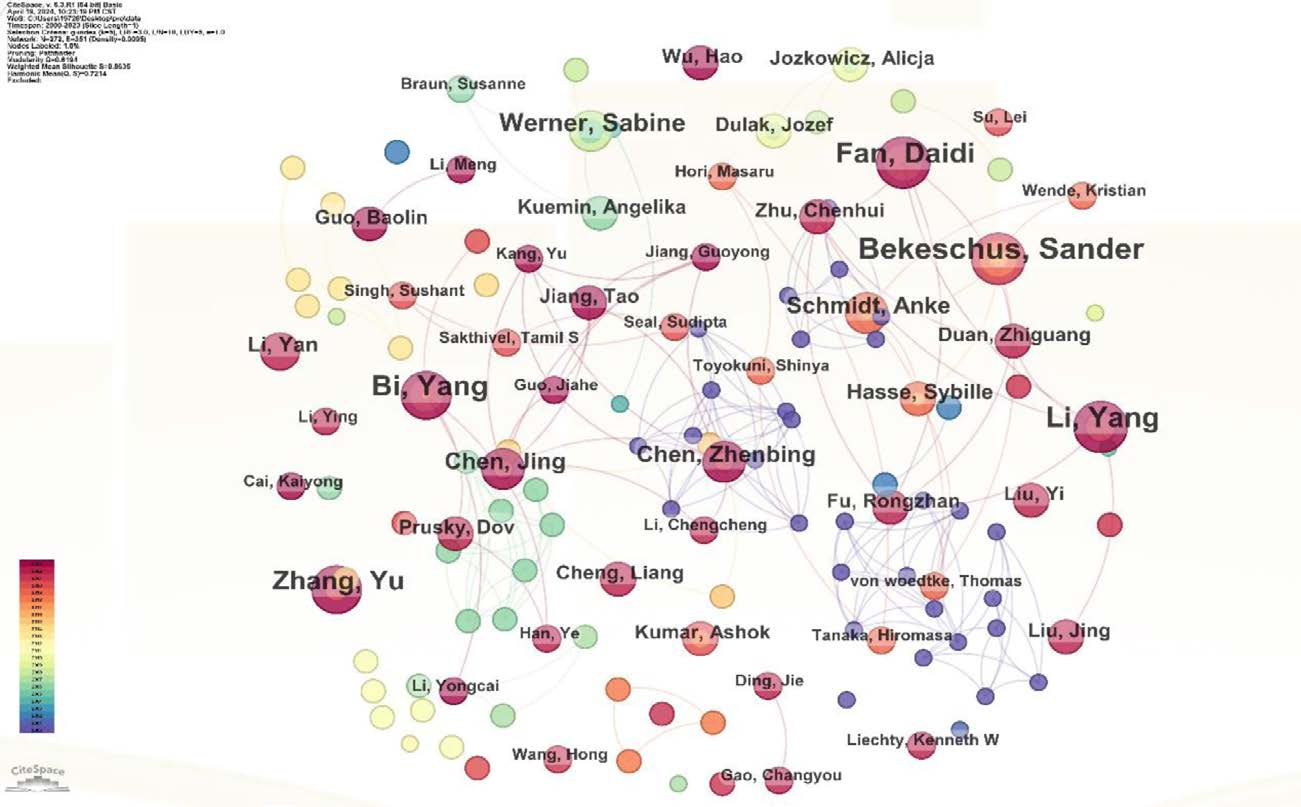
Author co-occurrence: Branches indicate that the author appears in the same article as other authors.

**Figure 3.**
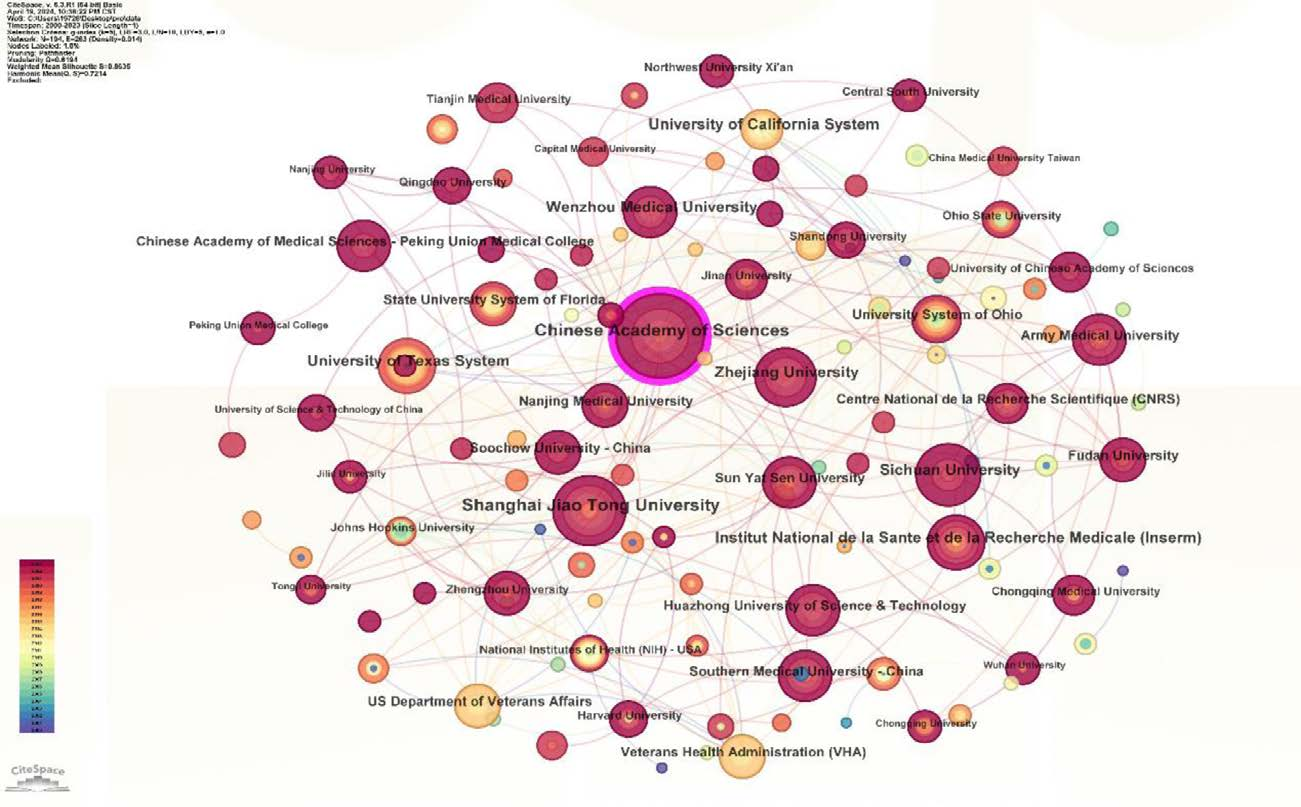
Organization co-occurrence 2000–2022.

**Figure 4.**
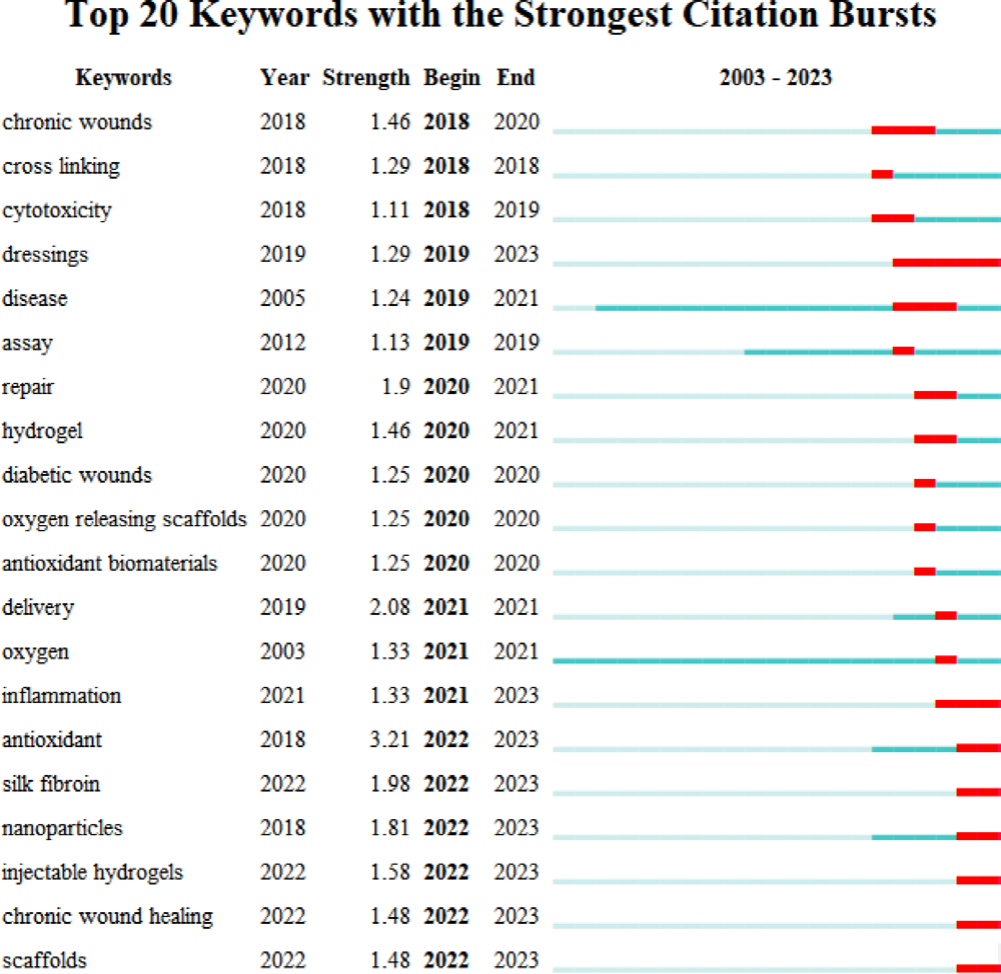
Dressing related keywords outburst time and intensity display.

**Figure 5.**
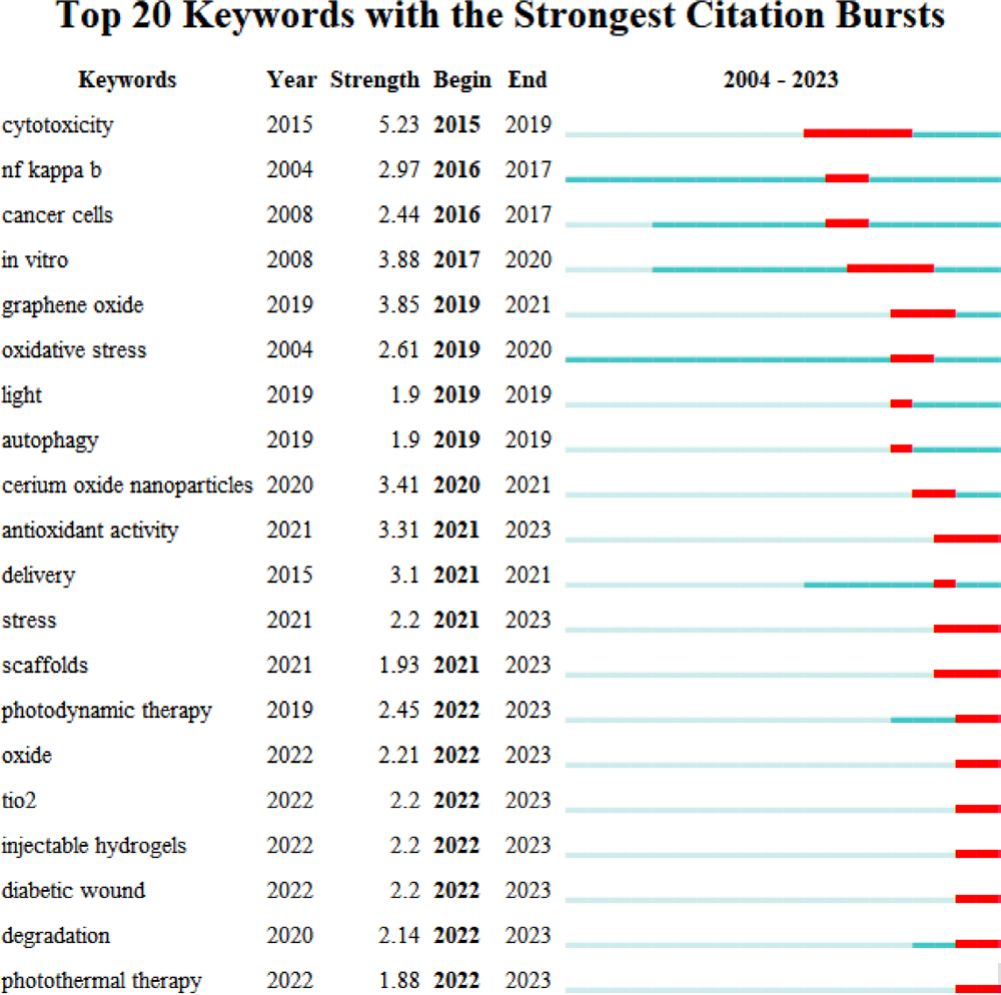
Display of key words related to the eruption time and intensity of nanomolecular materials.

By generating a reference co-citation map (Fig. 1), author co-occurrence map (Fig. 2), institution co-occurrence map (Fig. 3), burst word intensity display (Figs. 4 and 5), as well as presenting the most cited articles and journals in tabular form (Tables 1 and 2), we have gained a clear of the research trends in wound healing and oxidative stress. Additionally, we have conducted analyses on countries, institutions, authors, keywords, and more.

**Table 1:** Top 10 cited publications in the research on Oxidative Stress and Wound Healing from 2000 to 2022

No.	Title	First author	Journal	Citation	Year
1	Wound healing dressings and drug delivery systems: a review	Joshua S Boateng	Journal of Pharmaceutical Sciences	1858	2008
2	Applied plasma medicine	Fridman, Gregory	PLASMA PROCESSES AND POLYMERS	1548	2008
3	Curcumin: the Indian solid gold	Bharat B Aggarwal	Advances in Experimental Medicine And Biology	1217	2007
4	Antibacterial anti-oxidant electroactive injectable hydrogel as self-healing wound dressing with hemostasis and adhesiveness for cutaneous wound healing	Xin Zhao	Biomaterials	1199	2007
5	Regulation of matrix metalloproteinases: an overview	Sajal Chakraborti	Molecular And Cellular Biochemistry	921	2003
6	Hyaluronan fragments: an information-rich system	Robert Stern	European Journal of Cell Biology	819	2006
7	Cathelicidins, multifunctional peptides of the innate immunity	Margherita Zanetti	Journal of Leukocyte Biology	782	2004
8	The nuts and bolts of low-level laser (light) therapy	Hoon Chung	Annals of Biomedical Engineering	756	2012
9	Epub 2006 Apr 4. LL-37, the only human member of the cathelicidin family of antimicrobial peptides	Ulrich H N Dürr	Biochimica et Biophysica Acta	736	2006
10	Adhesive hemostatic conducting injectable composite hydrogels with sustained drug release and photothermal antibacterial activity to promote full-thickness skin regeneration during wound healing	Yongping Liang	Small	719	2019

**Table 2:** Top 5 most cited journals and Top 5 most publications in the research from 2000 to 2023

Most cited	JOURNAL OF ETHNOPHARMACOLOGY	5.4	Q1
	WOUND REPAIR ANDREGENERATION	2.9	Q2
	INTERNATIONAL JOURNAL OF BIOLOGICAL MACROMOLECULES	8.2	Q1
	INTERNATIONAL JOURNAL OF MOLECULAR SCIENCES	5.6	Q1
	PLOS ONE	3.7	Q2
	BIOMATERIALS	14	Q1
	ACS APPLIED MATERIALS & INTERFACES	9.5	Q2
	INTERNATIONAL JOURNAL OF BIOLOGICAL MACROMOLECULES	8.2	Q1
	INTERNATIONAL JOURNAL OF MOLECULAR SCIENCES	5.6	Q1
	PLOS ONE	3.7	Q2

### 3.1. Analysis of co-cited reference: clusters of research and most cited papers

Within this citation network, we have identified 19 distinct clusters and discovered three major research trends concentrated in three different time periods. These trends have been observed to evolve over time, with an overall shift towards the translational aspect from basic research to clinical applications (Fig. 1).

The first and most important trend is the molecular biology of wound healing, which started with three research clusters from 1995 to 2011. These clusters, labeled as cluster #1 (“nitric oxide”), #8 (“molecular biology”), and #17 (“melatonin”), have average publication years, representative references, and cluster members. These clusters further evolved into cluster #3 (“nitric oxide nanoparticle”), #7 (“skin myofibroblast differentiation”), #10 (“adipose-derived stem cell”), and #15 (“5 key factor”). This trend describes the gradual shift from characterization of wound repair towards exploring specific mechanisms in the field.

The second major trend of the research (2003–2016) is the integration of basic experiments with clinical studies. Clusters #9 (“plasma application”) and #14 (“healing promoter”) represent this trend. They further developed into clusters #18 (“healing promoter”), #19 (“foxo1 tgf-beta regulation”), and #6 (“physicochemical characteristics cell culture”). Around 2016, these clusters transformed into cluster #11 (“potential skin wound”) and #16 (“periodic exposure”). During this period, research on wound healing mechanisms has gradually improved, and the exploration of basic experiments has shown some initial effectiveness, with the start of experiments in clinical applications.

The third prominent trend in wound healing research is the application of diverse methodologies in clinical settings, commencing from 2012 to the current timeline. Initially, the clustering related to skin wound classification (cluster #2) and the shape memory property (cluster #14) transitioned into the multifunctional hydrogel cluster (#5). Presently, these have evolved into the recent advance (cluster #4) and the promotion of wound healing (cluster #0) clusters.

### 3.2. Most cited papers

In Table 1, the top 10 most cited references are listed. The most highly cited publication is a review article by Joshua S. Boateng et al, featured in the Journal of Pharmaceutical Sciences in 2008, with a remarkable 1858 citations. This article elucidates the advantages of integrating cutting-edge technologies with wound dressings to advance wound healing research, such as the favorable delivery properties of hydrogel dressings in conjunction with drug formulations.

The second and third most cited articles are “Applied plasma medicine” by Fridman, Gregory et al published in Plasma Processes and Polymers with 1548 citations, and “Curcumin: the Indian solid gold” by Bharat B Aggarwal et al in Advances in Experimental Medicine and Biology with 1217 citations. These papers delve into the formidable antioxidant effects of plasma medicine and curcumin, respectively.

**Figure 6.**
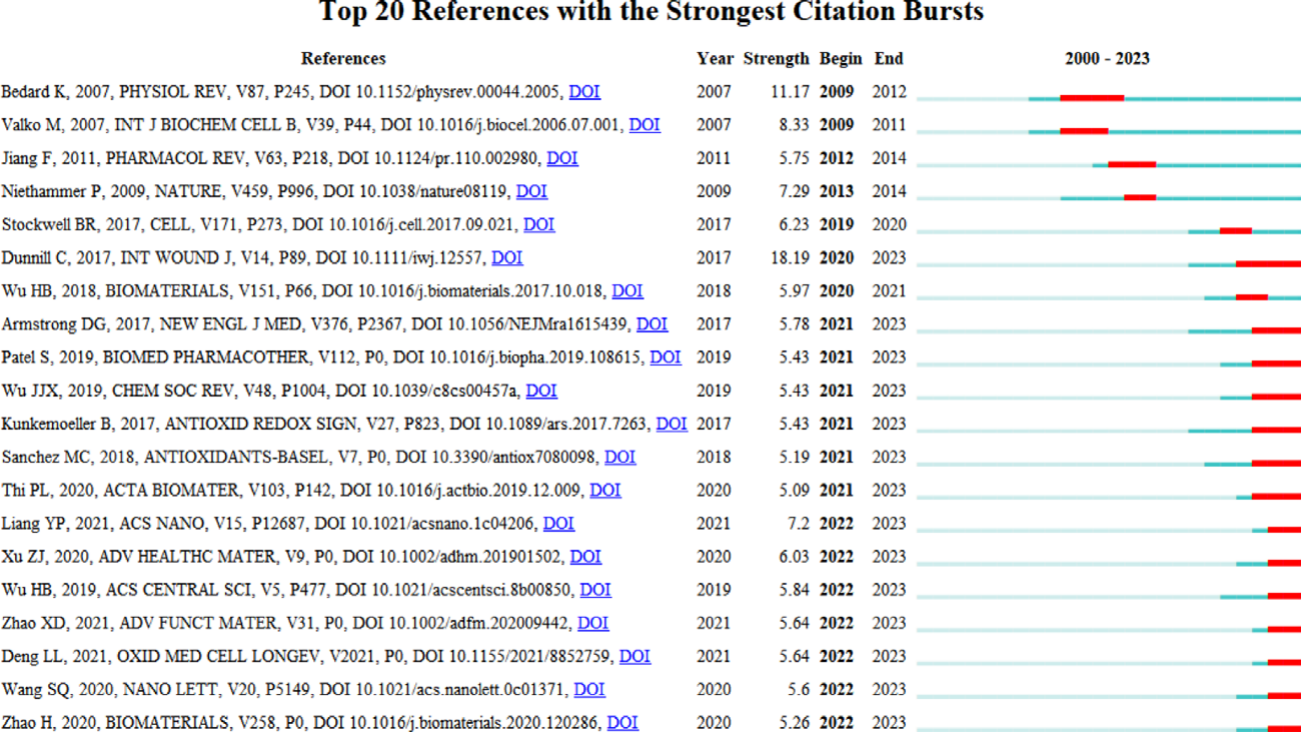
Top 25 papers with the strongest citation bursts.

Upon analyzing the sudden surge of references (Fig. 6) in literature, the top three most cited references across the whole span of years revolve around the correlation between nitric oxide and wound healing.

The foremost paper, “The function of nitric oxide in wound repair: inhibition of inducible nitric oxide-synthase severely impairs wound reepithelialization” by B Stallmeyer et al., published in the Journal of Investigative Dermatology in 1999, delves into the impact of nitric oxide synthase on the reepithelialization role of keratinocytes.

The second paper, “Nitric oxide triggers enhanced induction of vascular endothelial growth factor expression in cultured keratinocytes (HaCaT) and during cutaneous wound repair” by S Frank et al., featured in the Faseb Journal in 1999, elucidates the augmentation of vascular endothelial growth factor expression by keratinocytes upon exogenous nitric oxide addition, facilitating wound healing.

The third paper, “Reversal of impaired wound repair in iNOS-deficient mice by topical adenoviral-mediated iNOS gene transfer” by K Yamasaki et al., published in the Journal of Clinical Investigation, underscores the beneficial impact of nitric oxide synthase on wound healing.

In the analysis conducted within the past five years, the top three most cited references showcasing sudden bursts are all review articles. These include “Wound repair and regeneration: mechanisms, signaling, and translation” by Sabine A Eming et al., published in Science Translational Medicine, “Challenges in the Treatment of Chronic Wounds” by Robert G Frykberg et al, featured in Advances in Wound Care, and “The chemistry and engineering of polymeric hydrogel adhesives for wound closure: a tutorial” by C Ghobril et al, published in Chemical Society Reviews. These publications primarily discuss the advancements in wound dressing application in wound management, indicating that the current research trends are steered by review articles, with researchers employing literature compilation and analysis to reveal novel burgeoning research avenues.

### 3.3. Co-occurring keywords networks

By studying the changes in keywords, it has been found that research hotspots also change over time.

**Figure 7.**
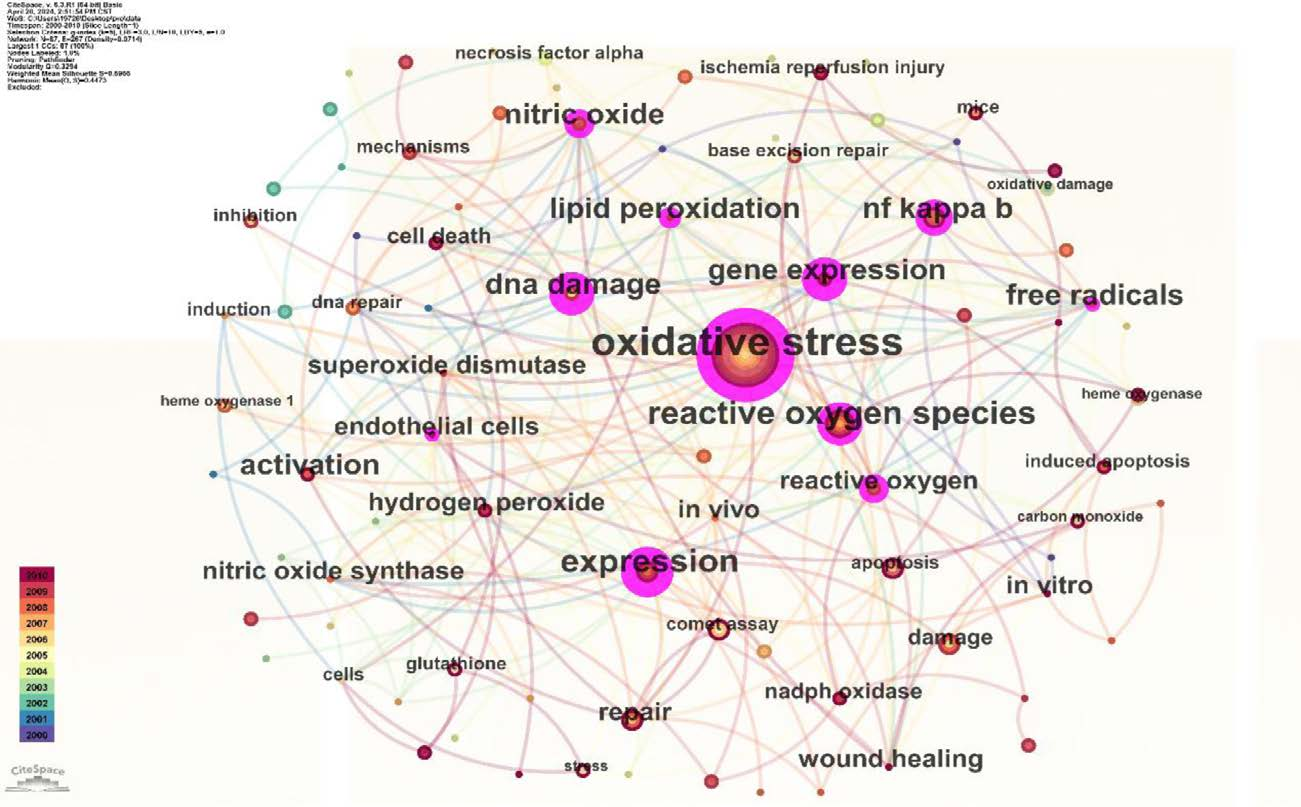
Cluster analysis of the keywords 2000–2010.

**Figure 8.**
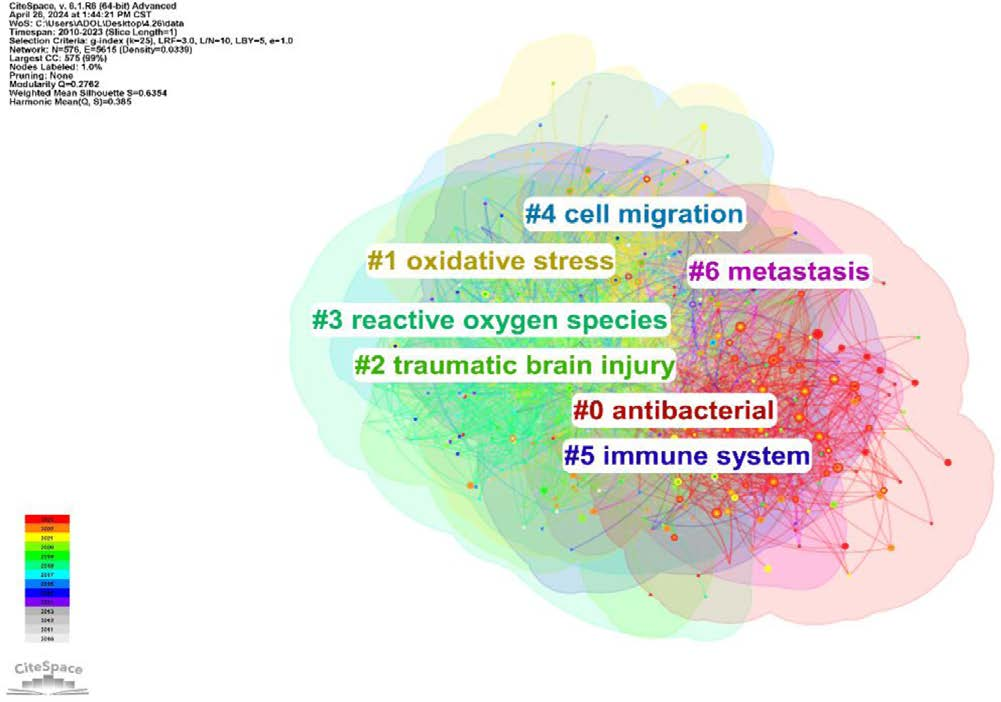
Cluster analysis of the keywords 2010–2022.

By analyzing the most frequently cited keywords, we can examine research hotspots and trends. We used CiteSpace to extract a timeline of co-occurring keywords from literature (2000–2023). Eight groups of keywords were identified (Fig. 7), with the most important ones being “oxidative stress,” “reactive oxygen species,” “expression,” “oxidative stress,” and “diabetic foot ulcer.” We further extracted the same network, focusing on the past 10 years (2012–2023) due to the timeliness of the articles (Fig. 8). Seven clusters were identified, with the most important cluster being “antibacterial,” followed by “oxidative stress,” “traumatic brain injury,” “reactive oxygen species,” “cell migration,” “immune system,” and “metastasis.”

**Figure 9.**
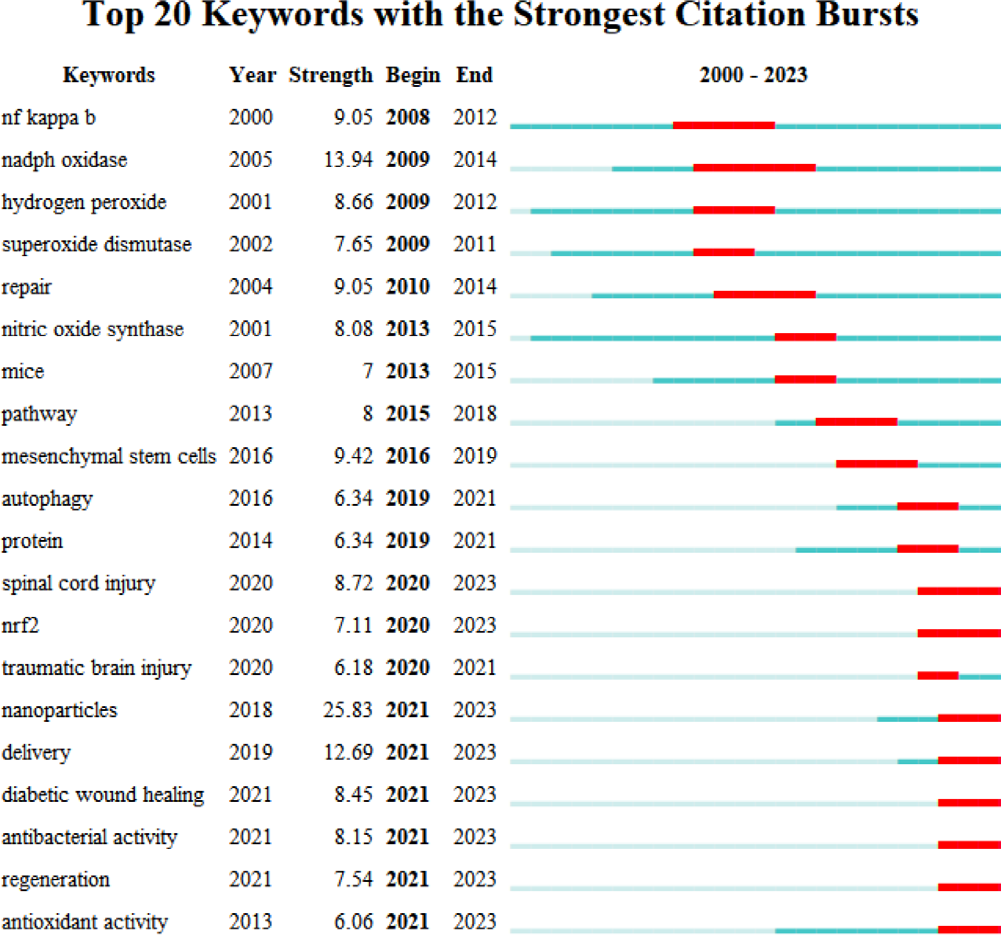
Top 25 keywords with the strongest citation bursts.

Additionally, when considering the time period of 2012 to 2023 (Fig. 9), the keywords with the latest citation bursts reveal the newest keyword trends, namely “nanoparticles,” “nadph oxidase,” and “delivery.” The changes in keywords reflect the changes in research hotspots.

### 3.4. Publication outputs and major journals

The initial dataset encompassed 3025 references. A meticulous evaluation of all 82 highly cited articles identified by WoSCC verified their relevance to the targeted subject. The final dataset comprised 3025 studies published between 2000 and 2023, comprising 2586 articles and 439 reviews. On average, each publication listed 5.40 authors, hailing from 133 diverse literary sources. The earliest discovered article was authored by Muscara, MN and colleagues, focusing on the effects of an NO-NSAID and a selective COX-2 inhibitor on mouse wound collagen fiber deposition.

The scientific productivity concerning the impact of oxidative stress on wound healing has been progressively escalating annually, notably from 2000, with an average annual growth rate of 19.96%. The publication count initiated at 15 publications per year in 2000 and exhibited exponential expansion, culminating in 529 publications in 2023, particularly surging post-2015. The mean number of citations per publication per year surged from 0.4 in 2000 to 41.6 in 2023.

The top 5 journals with the highest number of published articles were JOURNAL OF ETHNOPHARMACOLOGY (n = 60), WOUND REPAIR AND REGENERATION (n = 56), INTERNATIONAL JOURNAL OF BIOLOGICAL MACROMOLECULES (n = 52), INTERNATIONAL JOURNAL OF MOLECULAR SCIENCES (n = 52), and PLOS ONE (n = 39) (Table 2).

### 3.5. Analysis of cooperation networks across countries and institutions

**Figure 10.**
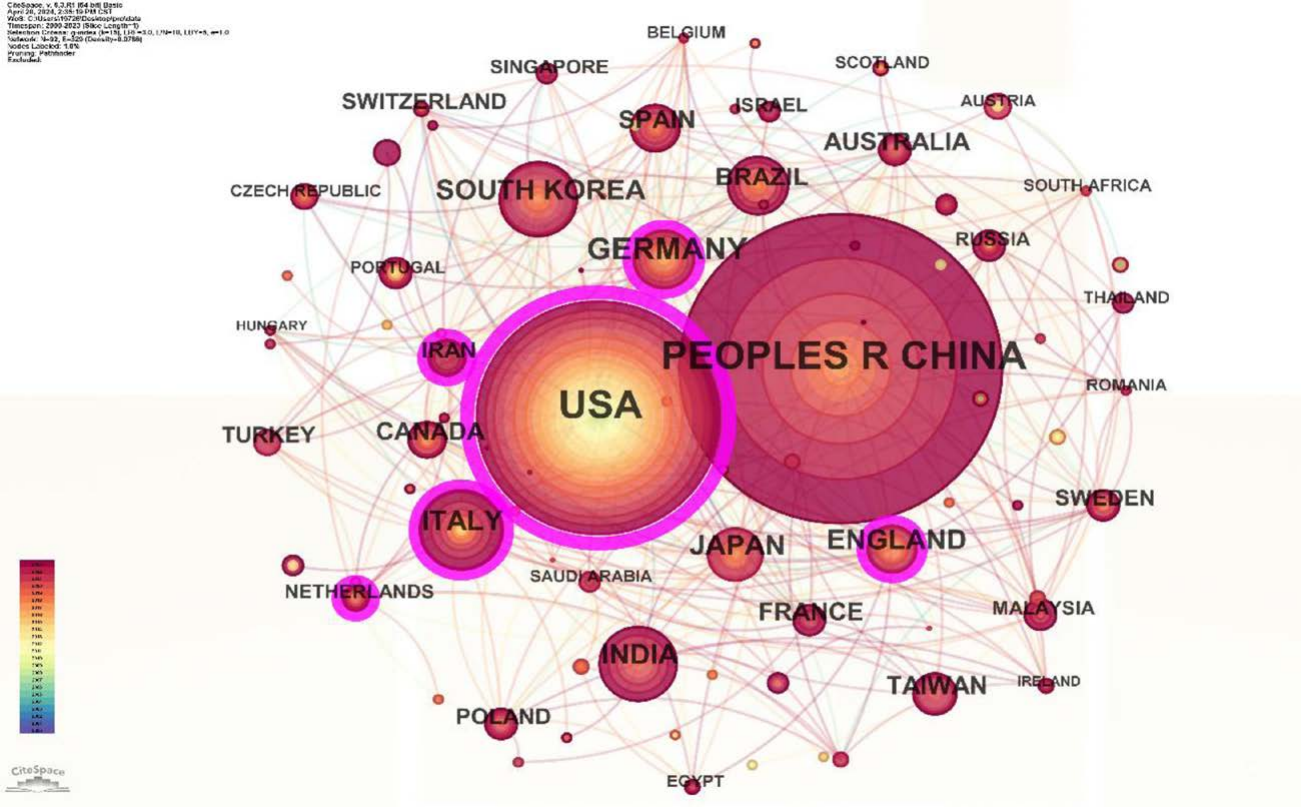
Map of countries and regions with publications from 2000 to 2022.

A total of 100 countries have been identified. Among them, CHINA has close connections with other nodes (Fig. 10) and has the highest centrality, indicating the highest level of collaboration with other countries. The next countries with high centrality are USA, ITALY, and ENGLAND.

**Figure 11.**
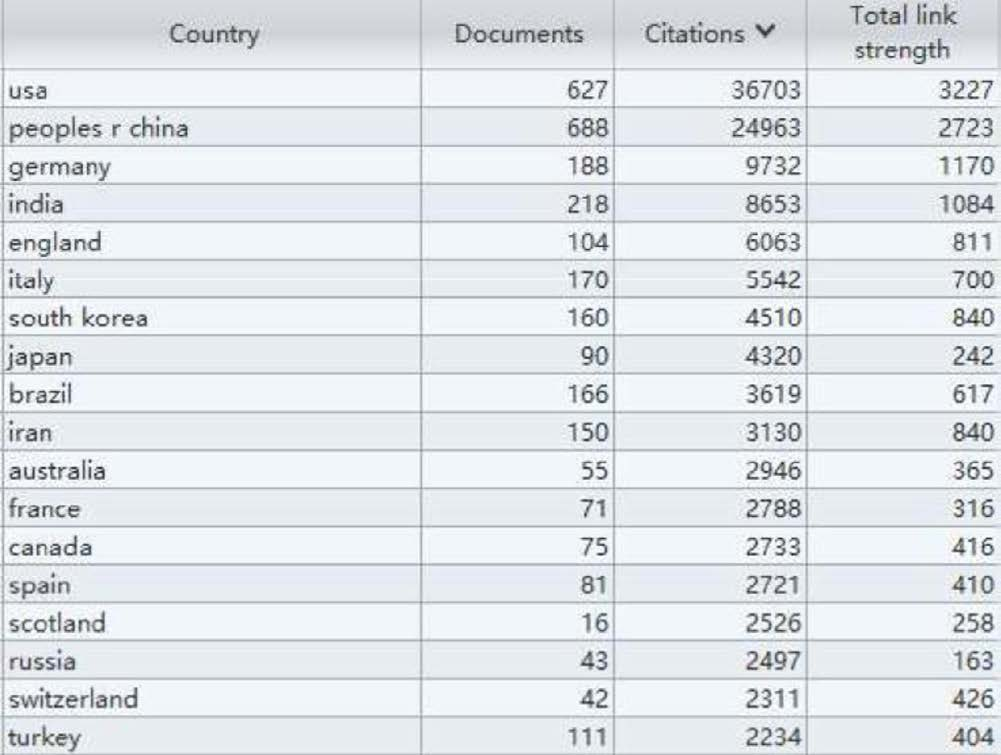
Top 10 countries and regions involved in the research from 2000 to 2022.

According to our database, the most cited country is the United States (n = 36,703), followed by China (24,963), Germany (9732), India (8653), and England (6063) (Fig. 11).

We have identified a total of 685 different organizations. The top 5 organizations in terms of citation count are Chinese Academy of Sciences, followed by Shanghai Jiao Tong University, Sichuan University, Zhe Jiang University, and Wenzhou Medical University.

This visualization map allows us to observe the interconnections and the level of connections between the most important organizations with major hotspots (Fig. 3).

### 3.6. Analysis of co-authorship network

We have retrieved the authors with the highest number of publications related to oxidative stress response in skin injury repair from our database, as well as their collaboration networks (as coauthors of publications) (Fig. 2).

The top 5 authors with the highest citation count in the past 5 years are Bekeschus, Sander, Li, Yang, Bi, Yang, Fan, Daidi, and Zhang, Yu.

Summary of the main findings

This study utilized 2 different software packages to comprehensively outline the development of oxidative stress-related research in wound healing since 2000, including the main countries, institutions, authors, journals, hotspots, and trend characteristics.

We found that publications on oxidative stress in wound healing have exponentially increased since 2000, with over 500 articles published by 2023. These studies primarily focus on the following aspects:

1. Mechanistic studies on the relationship between wound healing and oxidative stress.
2. Combination of antioxidant substances with wound dressings.
3. Application of nanomaterials in wound dressings by modulating oxidative stress.

The United States has the highest citation count, while China has the highest number of publications. Chinese Academy Sciences in China has the highest citation count among institutions, and Sichuan University has the highest number of published research outcomes among universities. The top 5 most cited authors are Guo, Baolin; Liang, Yongping; He, Jiahui; Zhao, Xin; and Frank, S. The most cited journals are Biomaterials, ACS Applied Materials & Interfaces, Wound Repair and Regeneration, Journal of Ethnopharmacology, and Chemical Engineering Journal.

The retrieved citation reference network (2000–2023) describes coherent connections among 19 different clusters, revealing the evolutionary trend of oxidative stress-related research in wound healing from basic to clinical, gradually transitioning from fundamental theory to clinical practice research.

## 4. Discussion

### 4.1. Identification of trends and future of evidence synthesis

A comprehensive bibliometric review of wound healing treatment literature spanning from 2000 to the conclusion of 2023 has revealed a notable trajectory: the transition of advancements from basic experimental research towards clinical application. An emerging research focal point lies in the integration of antioxidant compounds, wound dressings, and nanomaterials to enhance delivery efficiency.

In the recent 5-year period, scholarly attention has shifted towards investigating clinically applicable healing materials like oxidized konjac glucomannan, antibacterial materials, drug delivery systems, bacterial cellulose, electrospun nanofibers, injectable hydrogels, and zinc oxide nanoparticles. Research emphasis has also been directed towards mitigating common wound healing challenges, notably in diabetic wound healing.

These trends are predominantly discerned through sudden keyword statistics, which aid in pinpointing prevailing research tendencies, encompassing evidence synthesis (guidelines, reviews, meta-analyses) and keywords aligned with recent research progress (drug delivery systems, zinc oxide nanoparticles, diabetic wound healing). Notably, an examination of the timeliness aspect reveals that the most frequently cited articles in the past 5 years predominantly comprise meta-analyses and consensus guidelines. Additionally, the co-authorship keyword network portrays a surge in the quantity of network meta-analyses.

Through foundational research, it has been unraveled that wound healing entails not only hemostasis but also the recruitment of diverse cellular components within the skin to facilitate the process. Neutrophils, being the initial cells to reach the wound site, play a pivotal role in the healing cascade. Inadequate neutrophil recruitment poses infection risks and triggers delayed wound recovery, whereas excessive recruitment can instigate tissue damage and further impede healing. Neutrophils can release reactive oxygen species (ROS) during phagocytosis, serving the dual purpose of microorganism eradication and oxygen consumption, consequently establishing a hypoxic milieu in the afflicted region. This oxygen-depleted environment exacerbates the delay in wound healing, underscoring the significance of antioxidant therapy in mitigating delayed wound recovery.

In the realm of clinical investigations, hyperbaric oxygen therapy (HBOT) has emerged as a promising intervention for chronic wound treatment. Notably, the characteristic excess oxidative stress in wounds is attributable to inadequate oxygen provision, leading to variable degrees of tissue and area hypoxia. In such instances, HBOT serves as an efficacious adjunctive treatment avenue. By significantly elevating dissolved oxygen levels in the plasma, HBOT enhances tissue oxygenation, thereby catalyzing the healing process of unresponsive wounds under standard clinical care. HBOT augments oxygen tension, thereby meeting the energy demands of healing and diminishing infection incidence, accentuating the pivotal role of antioxidants in chronic wound treatment.

Antioxidants have emerged as pivotal constituents within local treatment strategies, offering promising avenues for enhanced wound management. The utilization of bibliometric analysis, inclusive of literature scrutiny in designated domains, has made notable strides in evaluating research hotspots and forecasting potential breakthroughs through mathematical and statistical approaches. In this context, the seamless integration of tools such as CiteSpace, VOSviewer, PubMed, and Web of Science has enabled the visualization of extensive literature data, signifying paramount importance in advancing this field of study.

### 4.2. Current outcomes on treatment of oxidative stress at wound sites

The cornerstone of wound antioxidant therapy lies in curbing the generation of peroxides and superoxide anions spurred by oxidative stress. Key therapeutic strategies in this domain encompass the localized application of antioxidant medications, the utilization of wound dressings, and their collaboration with nanomaterials.

#### 4.2.1. Crucial role of oxidative stress in wound healing

Wound healing embodies a complex biological and macromolecular cascade.^[18,25,26]^ The rigorous wound environment, characterized by enzyme imbalances, pH fluctuations, and a continuous outflow of free radicals - considered instigators of wound site inflammation - can culminate in delayed wound and scar healing.^[27]^ Hence, endeavors have been made to counteract oxidative stress processes and products by mitigating the overexpression of oxidizing agents such as lipid peroxides and superoxide anions within the wound-healing microenvironment. This overexpression extends the wound healing timeline; at heightened concentrations, these agents inflict tissue harm and cellular demise, impeding the natural healing progression. The inhibition of excessive agent production harbors therapeutic potential in wound management as it obstructs oxidative stress pathways, delivering anti-inflammatory and additional effects.

Antioxidant therapy revolves around nurturing a conducive wound microenvironment and furnishing antioxidants, which can be dispensed via wound dressings. Antioxidants, broadly categorized into enzymes (e.g., superoxide dismutase (SOD)^[28]^) and non-enzymatic antioxidants (e.g., vitamins, polyphenols), are pivotal elements in this therapeutic strategy owing to the inherent fragility and susceptibility of enzymes to inactivation.^[29]^ Correspondingly, researchers have pivoted towards non-enzymatic antioxidants for investigation. Common antioxidants amenable to incorporation into dressings encompass the following compounds.

##### 4.2.1.1. Vitamins

Vitamin E primarily localizes on the cell membrane, shielding it from the detrimental effects of reactive oxygen species that could harm the cell membrane lipids.^[30]^ On the other hand, Vitamin C plays a vital role in reducing oxidized vitamin E in the cytoplasm. Moreover, when encountering active oxygen species like hydrogen peroxide within cells, Vitamin C can neutralize and eliminate some of these species. Given that vitamins are essential nutrients, individuals readily embrace oral vitamin supplementation. Recognizing the multifaceted benefits of vitamins beyond antioxidant properties, such as overall well-being enhancements, individuals often employ vitamin supplements alongside other topical antioxidants. Common antioxidants that can be seamlessly integrated into wound dressings encompass the following compounds.

##### 4.2.1.2. Polyphenol antioxidants

Polyphenols, distinguished by a molecular weight exceeding 600 Da and featuring multiple phenolic hydroxyl groups as seen in compounds like curcumin, resveratrol, and catechins, are predominantly obtained from plant sources. The antioxidant prowess of polyphenols is intricately linked to their chemical composition.^[31]^ Acting as potent nucleophiles, polyphenolic compounds leverage the electron-donating propensity of their hydroxyl groups; however, they are susceptible to oxidation through electron loss. Furthermore, the involvement of hydrogen atoms within the phenolic hydroxyl groups is critical in biochemical reactions.^[32]^ These hydrogen atoms can be emitted either as protons under basic conditions or as hydrogen radicals, leading to the formation of phenolate anions and phenoxyl radicals, which undergo reciprocal electron exchange. Phenoxyl radicals exhibit stability due to resonance effects, enabling them to effectively scavenge free radicals by combining with other radicals, thus manifesting their antioxidant properties.

1. Curcumin
  Curcumin, derived from turmeric, is a natural polyphenolic compound^[33]^ known for its ability to stimulate the formation of granulation tissue, accelerate wound contraction,^[34]^ and exhibit anti-inflammatory, antibacterial, and antioxidant properties. Notably, in the top three most cited papers of the past two decades, both the second and third papers are centered on curcumin (Table 1). Jonathan G. Merrell et al demonstrated that curcumin treatment on rat skin wounds led to decreased levels of peroxides, such as lipid peroxides, while increasing the activity of peroxide-scavenging enzymes like SOD, catalase, and glutathione peroxidase.^[35]^ Despite its potent antioxidant capabilities, curcumin faces challenges with low bioavailability, hindering its effective delivery to wound sites through oral administration. Its hydrophobic nature and poor penetration further limit its efficacy in neutralizing free radicals.^[36]^ To combat these limitations, direct application of curcumin onto wounds is advocated, aiming to overcome issues related to its poor solubility and dissolution rates, thus enhancing drug effectiveness. Incorporating curcumin into nanostructured materials emerges as a promising strategy to address these challenges. The nano-fiber architecture resembles the extracellular matrix, offering a high surface-volume ratio, porosity, and enhanced oxygen and gas permeability, which optimize its antioxidative potential.^[34]^ Additionally, Amrita Kumari et al explored the application potential of curcumin and nano-carrier polymers in wound healing, highlighting hydrogels as the preferred carrier system.^[8]^
2. Resveratrol
  Resveratrol, also known as 3,5,4′-trihydroxy-trans-stilbene, is a natural polyphenol present in red wine and grapes. Classified under the stilbenes group of polyphenols, resveratrol demonstrates anticancer, anti-scarring, and antioxidant properties. Its antioxidative efficacy stems from its capacity to scavenge oxygen free radicals and regulate antioxidant pathways.^[37]^ In a study by Orihuela-Campos et al, it was observed that a concentration of 50 µM resveratrol could impede the production of reactive oxygen species (ROS).^[38]^ Resveratrol plays a pivotal role in modulating the Sirt1 and AMPK pathways, leading to the activation of peroxisome proliferator-activated receptor gamma coactivator-1 alpha. Clinical application of resveratrol in treating diabetic foot ulcers involving 45 patients resulted in a twofold enhancement in wound healing capabilities and a reduction in oxidative stress levels.^[39]^ Notably, research indicates that resveratrol influences sirtuin 6, a member of the sirtuin family, initiating macrophage adhesion and migration responses that promote effective wound recovery.^[40]^ Furthermore, resveratrol has been found to facilitate M2 macrophage polarization, diminishing the production of pro-inflammatory cytokines prompted by oxidative stress, thereby expediting the wound healing process.^[41]^
3. TP (tea polyphenol)
  Tea polyphenols encompass the collective polyphenolic compounds present in tea, such as catechins, flavonols, flavones, flavanols, and phenolic acids.^[42]^ These polyphenols exhibit potent antioxidant characteristics, effectively scavenging free radicals within the body.^[43]^ They impede lipoxygenase activity and lipid peroxidation in skin mitochondria. Studies reveal that 1 milligram of tea polyphenols possesses a free radical-clearing efficacy equivalent to 9 micrograms of SOD, surpassing the potency of other similar substances significantly. The antioxidant prowess of tea polyphenols surpasses that of vitamin E by 18 times and complements the actions of vitamins C and E synergistically.^[44]^ In a study by Hangye Zhao et al, the use of ImageJ software to determine the average optical density of tea polyphenols indicated a decline in catalase activity, reinforcing the antioxidative impact of these compounds.^[45]^

##### 4.2.1.3. Other antioxidants

Carotenoids like β-carotene (138 papers), lycopene (77 papers), and astaxanthin (64 papers), along with soy peptides (11 papers), silver (20 papers), and various other natural antioxidants, function as hydrogen donors to combat free radicals and hinder oxidative processes at the wound site.

#### 4.2.2. Wound dressings containing antioxidants are also an important new way to promote wound healing

**Figure 12.**
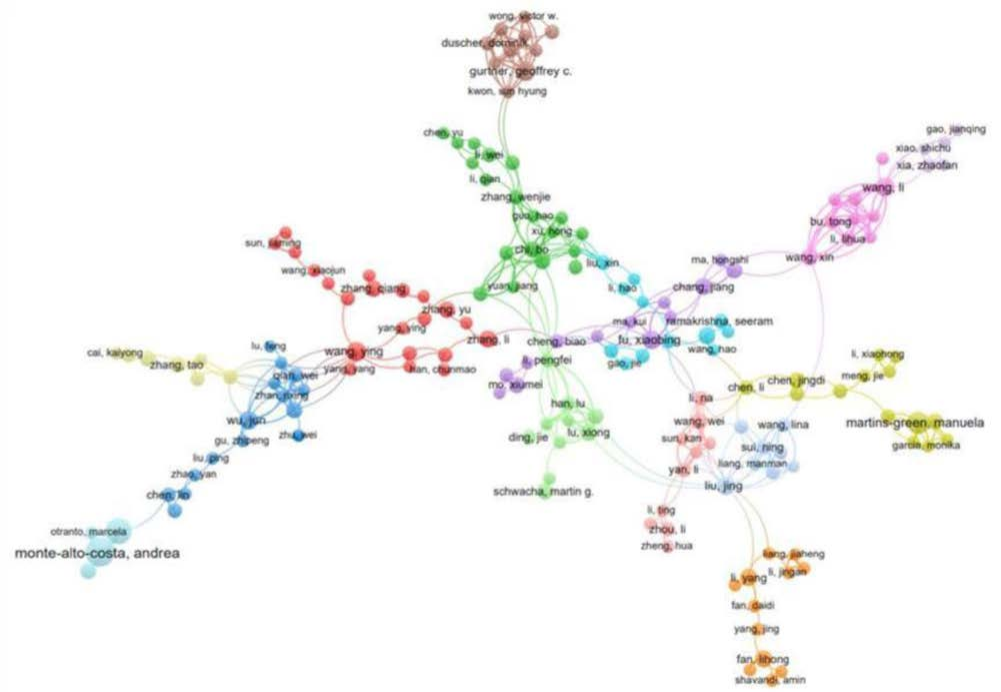
Analysis of authors and co-cited authors in the research.

Using VOSViewer (Fig. 12), we further extracted the visualization overlay of coauthor keywords network based on the average publication year spanning from 2000 to 2023. Notably, the most referenced and contemporary research trends were identified by keywords such as “wound healing” and “wound dressing.”

Subsequently, focusing on “wound dressing” within the literature results, 46 relevant papers were found. By setting the duration to 1, 15 burst words were obtained. Among these, key burst intensity keywords included “antioxidant” (intensity: 3.21), “delivery” (intensity: 2.08), “silk fibroin” (intensity: 1.98), “repair” (intensity: 1.9), and “nanoparticles” (intensity: 1.81). Excluding these five keywords, the most prevalent type of dressing directly referenced in the literature was “hydrogel” with a burst intensity of 1.46, observed throughout the year 2020 (Fig. 4). Notably, in that year, a total of 31 related publications were identified, with the most cited article being “Photo-responsive supramolecular hyaluronic acid hydrogels for accelerated wound healing” by Weiyi Zhao et al, delving into the benefits of supramolecular hydrogels for controlled drug delivery.

Hydrogel dressings represent an innovative form of wound dressing comprising highly absorbent polymer gels with elevated water content. Studies have exhibited their superior efficacy in promoting wound healing compared to traditional dry dressings. Additionally, their transparency allows for direct observation of the wound and its progression. The integration of antioxidant therapy with hydrogel dressings presents a promising avenue in the realm of wound recovery, enhancing wound healing capacities by preventing moisture loss and bacterial incursion.

Zhenghao Wang et al incorporated interleukin-33, an antioxidant, into a hydrogel dressing, showcasing no adverse effects on cell viability while fostering rapid clearance of reactive oxygen species (ROS).^[46]^ In a parallel endeavor, Huanhuan Chen et al devised a microbial hydrogel dressing incorporating live bacteria, effectively mitigating reactive oxygen species.^[47]^ These innovative methodologies harnessing the body’s natural defense mechanisms for wound treatment offer prospective benefits over traditional surgical and medicative modalities. Natural antioxidants, easily accessible and preferred, offer a novel, less side-effect-laden approach to antioxidant therapy, particularly beneficial for chronic non-healing wounds resistant to conventional treatments.

Exploring the attributes of natural antioxidants like curcumin, Nazli Seray Bostanci et al encapsulated curcumin into hydrogels comprising PeMA and GelMA at varying v/v ratios (1:1, 1:2, and 1:3) to evaluate wound healing efficacy across different pH levels. Remarkably, the curcumin-loaded P1:G3 hydrogel exhibited promising applications in treating infected and chronic wounds.^[48]^ Integrating antioxidants into wound dressings not only boosts wound healing capabilities and prevents moisture loss and bacterial intrusion but also propels innovative strategies for managing chronic wounds. Nonetheless, challenges persist, including the low solubility of antioxidants and inefficiencies in their delivery to the wound site. In contrast, supramolecular hydrogels emphasize chemical interactions between molecules, regardless of being organic or inorganic, whereas nanoparticles present robust catalytic activity in oxidation-reduction reactions due to their exceptional performance. Comprising inorganic metals or organic/inorganic nonmetallic materials, nanoparticles offer heightened delivery efficacy, aligning with the insights derived from the burst words data.

#### 4.2.3. The development of nanotechnology has promoted the progress of the relationship between oxidative stress and wound healing

In the past two decades, research articles focusing on nanotechnology’s impact on oxidative stress and wound healing have identified keywords of high significance, including nanoparticle, in vivo, release, mesenchymal stem cell, and inflammation. The findings suggest that nanoparticles possess targeting and slow-release capabilities, playing a vital role in the human body. Nanomaterials, defined as materials with at least one dimension below 100 nm or composed of fundamental structural units within this scale range, offer unique functionalities.^[49–51]^ The inherent size advantage of nanoparticles facilitates their efficient traversal across biological barriers.^[52]^ These nanoparticles not only function as antioxidants to enhance wound healing but also serve as carriers for antioxidant delivery, further promoting wound recovery. Particularly in wound dressings, nanoparticles demonstrate excellent permeability properties. Given that the skin serves as the body’s largest organ and presents a formidable barrier to large molecular particles due to its cuticle layer,^[53]^ nanoparticle delivery becomes a viable solution for therapeutic purposes. Several studies, such as that conducted by Nosheen Masood et al., have highlighted the efficacy of sustained-release nano-silver particles in treating chronic wounds, showcasing slow and consistent release properties over an extended period.^[54]^ Similarly, Caroline Tyavambiza et al emphasized nano-silver’s antioxidant and wound healing benefits attributed to its effective delivery mechanism.^[54]^ Antioxidant-rich natural materials have garnered considerable interest among researchers, as demonstrated by Qianmin Ou et al, who successfully integrated antioxidative herbs like puerarin into hydrogel nanodressings, resulting in enhanced antioxidant enzyme activities and reduced oxidative damage, ultimately promoting wound healing, tissue regeneration, and collagen accumulation.^[55]^ Additional studies, such as that by Ola Elkhateeb et al, revealed the enhanced antioxidant effects of curcumin when formulated as nanostructured lipid nanocarriers, displaying increased phenolic content and antioxidant activity compared to curcumin alone.^[56]^ Furthermore, research by Gerui Ren et al showcased the antioxidant properties of resveratrol nanoparticles (Z-R/H), capable of scavenging oxygen radicals while preserving the desirable attributes of peppermint oil through a unique adsorption mechanism, confirming improved antioxidant efficacy and delivery efficiency post-nanoparticle formulation.^[57]^ The amalgamation of localized treatments, such as combining nano-compress with antioxidant therapies, has shown promising outcomes in chronic wound management. Progressing from foundational laboratory investigations to clinical applications, this approach holds potential to advance chronic wound healing practices and contribute significantly to future research in the field.

### 4.3. Relevance of scientometric studies for evidence synthesis

**Figure 13.**
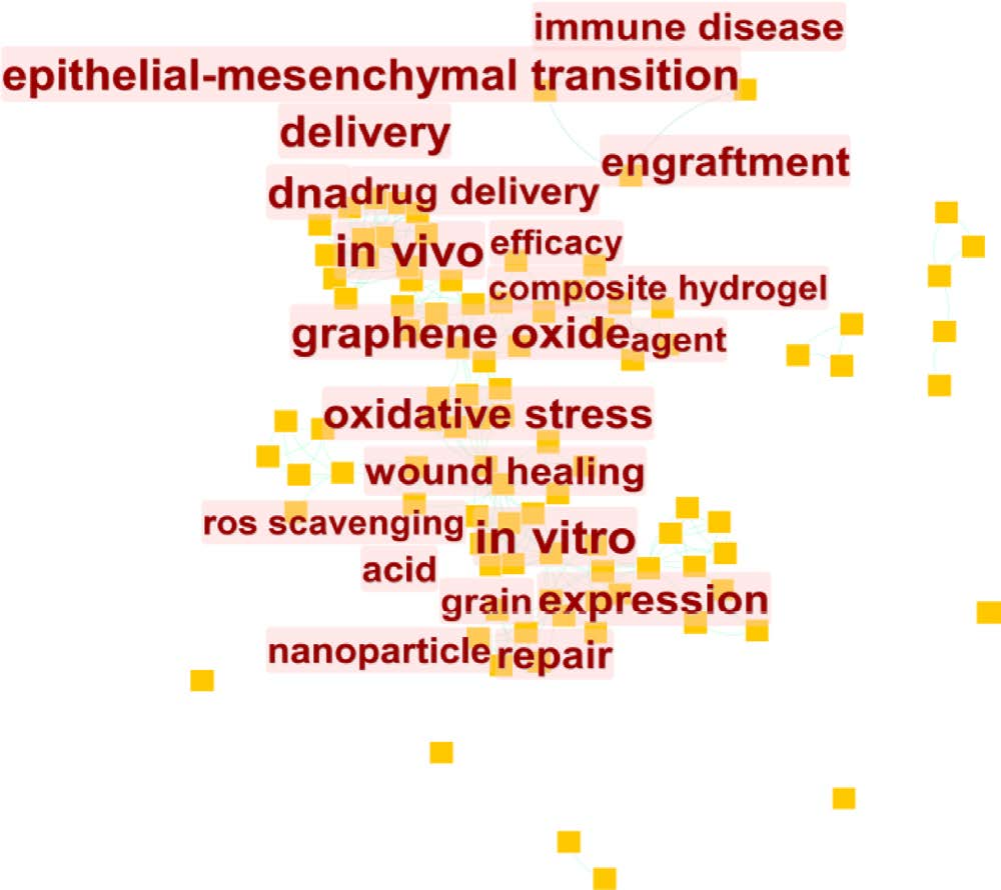
Keyword co-occurrence: Chinese Academy Sciences keyword co-occurrence.

**Figure 14.**
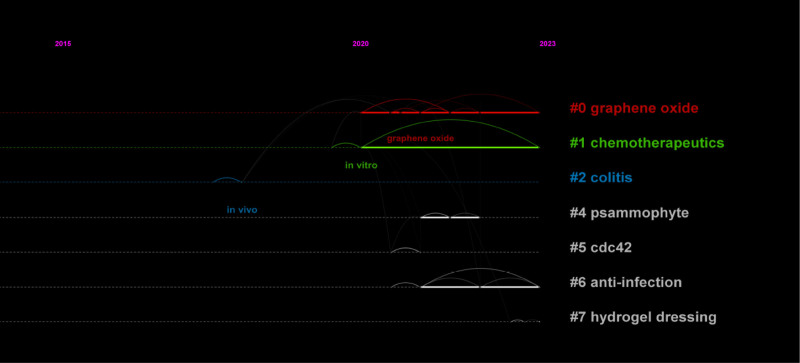
Chinese Academy Sciences clustering time map.

Furthermore, this research serves to identify key authors and journals within the field of oxidative stress in wound healing, offering valuable insights for junior researchers seeking mentors, institutions, stakeholders, decision-makers, and funding agencies, and guiding them on the current research trends. This study represents the inaugural scientometric investigation on oxidative stress in wound healing. Our analysis encompassed national and institutional affiliations, authors, keywords, and cited articles, leading to the following findings: From 2000 to 2023, Japan demonstrated the closest collaborative ties with other countries in terms of published articles. The United States emerged as the most frequently cited country, followed by China in second place. The Chinese Academy of Sciences stood out as the most cited institution. Leveraging this data, we performed clustering analysis and co-occurrence analysis of keywords associated with this institution (Refer to Figs. 13 and 14). Through these analyses, we identified top recurring keywords, such as oxidative stress (Refer to Fig. 14). Noteworthy clusters include #0 (graphene oxide, 2000), #1 (chemotherapeutics, 2020), #2 (colitis, 2020), signaling a progression in wound healing and oxidative stress research from molecular investigations to fundamental experiments and, more recently, to clinical applications.

### 4.4. Limitations

Scientometric analyses offer objective and comprehensive guidance for clinicians and scholars to discern research history and emerging trends. Additionally, they provide insight into clinically relevant questions that remain inadequately addressed, potentially shaping future experimental endeavors. However, limitations exist in scientometric studies, notably stemming from the reliance on citation-related metrics. These metrics are susceptible to biases, prominently citation bias, as the intent behind referencing is often to signify manuscript quality rather than true relevance or merit. This practice can lead to an underutilization of existing evidence. Various factors contribute to citation bias, including self-citations, author authority, journal impact factor, and the prestige of the publishing journal. Other biases, such as novelty bias, outcome reporting bias, location bias, and publication bias, can also affect scientometric research outcomes. Improving the quality of analysis is aided by incorporating homonymous authors. Publication bias manifests as a tendency to favor studies with significant results while discounting those with negative findings. The limitation of data sourcing solely from WoSCC may result in incomplete publication retrieval, underscoring the need to consider alternative databases like PubMed, Embase, and the Cochrane Systematic Review Database for a more expansive dataset. Even though WoSCC is a preferred database for scientometric research, future advancements in software may facilitate concurrent analysis of multiple databases with automated duplicate removal features, enhancing research outcomes. Notably, the co-citation network in this study predominantly focuses on first authors, potentially overlooking the full extent of author influence. Additionally, keyword merging methods, while reasonable, may benefit from refinement to enhance clustering accuracy. Addressing citation lag is crucial as recent publications may not yet be adequately cited, potentially affecting the detection of current trends.

## 5. Conclusion

This article applies bibliometric methods to objectively analyze research literature and academic output. By processing extensive literature data and validating the data and results through original sources, the study spans multiple disciplines and visualizes complex research data. These methods assist researchers in identifying potential opportunities for international cooperation and in fostering knowledge sharing on a global scale. Our investigation delved into the evolving trends in oxidative stress research within the context of wound healing. Through the examination of the co-citation network of relevant keywords, we identified the emerging treatment approach of utilizing antioxidants to promote wound healing. Subsequent research revealed that initiating treatment with specialized wound dressings is a novel strategy, with an emphasis on nanomolecular materials evident in recent clustering analyses. Exploring diverse natural antioxidants offers researchers additional avenues for exploration, aiding in the selection of appropriate dressings. Furthermore, the advancements in nanotechnology have accelerated progress in treatment strategies, particularly in enhancing delivery efficiency. Enhanced collaboration is recommended among key stakeholders in the United States, European institutions, and emerging influential entities in China. Our study furnishes valuable insights for researchers seeking a comprehensive view of the evolution of oxidative stress research in wound healing, providing essential guidance for researchers, funding applicants, agencies, and policymakers. Additionally, analyzing publications can assist scholars in identifying countries and journals, facilitating more meaningful academic exchanges.

Data presented in this study are available on request from corresponding author.

## Author contributions

**Conceptualization:** Fan Bu.

**Data curation:** Fan Bu, Li Rong.

**Formal analysis:** Fan Bu.

**Investigation:** Fan Bu, Kai Yu.

**Methodology:** Fan Bu.

**Project administration:** Fan Bu.

**Resources:** Fan Bu, Jinnan Wang.

**Visualization:** Qiaoyu Li.

## Supplementary Material
