# Supplementary figures and images for: Knowledge map and emerging trends of oxidative stress in wound healing: A bibliometric analysis from 2000 to 2023
Source: Medicine (Baltimore). 2025 Mar 7;104(10):e39970. doi: 10.1097/MD.0000000000039970 (PMC11902982; doi:10.1097/MD.0000000000039970)

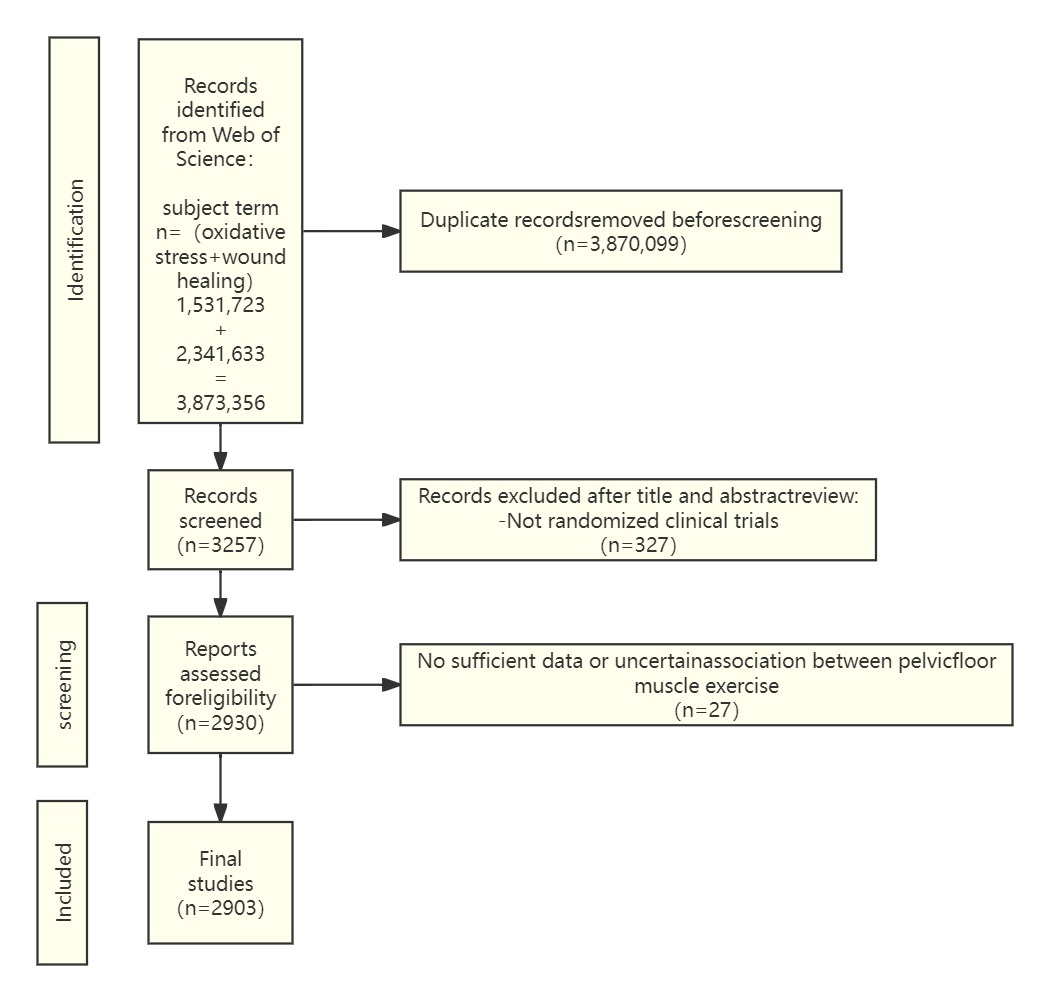


Supplementary Figure 1 ：Document screening flow chart

Supplement: Supplementary file 1 [file medi-104-e39970-s001.docx]
